# Toward Enhanced Clinical Decision Support for Patients Undergoing a Hip or Knee Replacement: Focus Group and Interview Study With Surgeons

**DOI:** 10.2196/36172

**Published:** 2023-04-24

**Authors:** Sabrina Grant, Emma Tonkin, Ian Craddock, Ashley Blom, Michael Holmes, Andrew Judge, Alessandro Masullo, Miquel Perello Nieto, Hao Song, Michael Whitehouse, Peter Flach, Rachael Gooberman-Hill

**Affiliations:** 1 Musculoskeletal Research Unit University of Bristol Southmead Hospital, Bristol Medical School Bristol United Kingdom; 2 Digital Health Faculty of Engineering Bristol United Kingdom; 3 Faculty of Medicine, Dentistry and Health University of Sheffield Sheffield United Kingdom; 4 Intelligent Systems Laboratory, Department of Computer Science University of Bristol Bristol United Kingdom

**Keywords:** arthroplasty, knee replacement, hip replacement, orthopedic surgery, clinical decision-making, postoperative follow-up, home monitoring, wearables, video

## Abstract

**Background:**

The current assessment of recovery after total hip or knee replacement is largely based on the measurement of health outcomes through self-report and clinical observations at follow-up appointments in clinical settings. Home activity-based monitoring may improve assessment of recovery by enabling the collection of more holistic information on a continuous basis.

**Objective:**

This study aimed to introduce orthopedic surgeons to time-series analyses of patient activity data generated from a platform of sensors deployed in the homes of patients who have undergone primary total hip or knee replacement and understand the potential role of these data in postoperative clinical decision-making.

**Methods:**

Orthopedic surgeons and registrars were recruited through a combination of convenience and snowball sampling. Inclusion criteria were a minimum required experience in total joint replacement surgery specific to the hip or knee or familiarity with postoperative recovery assessment. Exclusion criteria included a lack of specific experience in the field. Of the 9 approached participants, 6 (67%) orthopedic surgeons and 3 (33%) registrars took part in either 1 of 3 focus groups or 1 of 2 interviews. Data were collected using an action-based approach in which stimulus materials (mock data visualizations) provided imaginative and creative interactions with the data. The data were analyzed using a thematic analysis approach.

**Results:**

Each data visualization was presented sequentially followed by a discussion of key illustrative commentary from participants, ending with a summary of key themes emerging across the focus group and interview data set.

**Conclusions:**

The limitations of the evidence are as follows. The data presented are from 1 English hospital. However, all data reflect the views of surgeons following standard national approaches and training. Although convenience sampling was used, participants’ background, skills, and experience were considered heterogeneous. Passively collected home monitoring data offered a real opportunity to more objectively characterize patients’ recovery from surgery. However, orthopedic surgeons highlighted the considerable difficulty in navigating large amounts of complex data within short medical consultations with patients. Orthopedic surgeons thought that a proposed dashboard presenting information and decision support alerts would fit best with existing clinical workflows. From this, the following guidelines for system design were developed: minimize the risk of misinterpreting data, express a level of confidence in the data, support clinicians in developing relevant skills as time-series data are often unfamiliar, and consider the impact of patient engagement with data in the future.

**International Registered Report Identifier (IRRID):**

RR2-10.1136/bmjopen-2018-021862

## Introduction

### Background

Hip and knee replacements are major surgical procedures that aim to improve function and reduce pain related to joint diseases, particularly osteoarthritis. During hip or knee replacement, the affected joint is removed and replaced with an artificial joint. In 2019 in the United Kingdom, the National Joint Registry recorded 101,651 hip replacements and 108,713 knee replacements [1]; in the Unites States, >1 million total hip and knee replacement procedures are performed each year [2]. These surgical procedures are increasingly common, and numbers are projected to increase as a result of aging populations and increasing prevalence of obesity [3].

UK clinical guidelines for follow-up after hip and knee replacement surgery usually include face-to-face consultation, radiographs, and an assessment of health outcomes through telephone or web-based patient-reported outcome measures (PROMs) [4]. PROMs are designed to assess patients’ own views of their health and outcomes without interpretation by clinicians or others [5]. Of these, generic measures such as the 12-item Short Form Survey [6] and EQ-5D [7] aim to assess all important dimensions of health-related quality of life [8]. The Oxford Hip Score (OHS) [9] and Oxford Knee Score (OKS) [10] are additional disease-specific PROMs used by orthopedic surgeons in the United Kingdom. As validated instruments, PROMs are valuable sources of information for clinicians and researchers. However, several practicalities must be considered when implementing PROMs: missing or incomplete data; potential burden for patients; and cost, time, and administrative labor-intensiveness [11-13]. A recent review found that PROMs were prone to several types of bias: bias because of collection mode; nonresponse bias; proxy or caregiver response bias; recall bias (eg, bias because of the quality of patient recollection of past states); language bias (eg, semantic ambiguity); timing bias, representing a limited number of snapshots; and fatigue bias [13,14]. The OKS and OHS in particular may also fail to stratify activity level across a younger, more active population as they are not designed for this purpose. Instead, other instruments such as the Knee Injury and Osteoarthritis Outcome Score may be used, which are specifically designed for young and physically active patients, capturing additional domains of sport and recreation function and knee-related quality of life such that it has greater responsiveness as an outcome measure [15]. Taken together, these issues mean that, although PROMs are extremely valuable sources of information for clinicians and researchers, particularly because of their standardized and validated status, it is worthwhile to consider other methods to assess outcomes after joint replacement [16,17] that could be used in parallel to PROMs to support decision-making.

In this study, we used a qualitative approach to explore how time-based data may be used by clinicians to supplement PROMs. Qualitative methods were used to explore inductively what matters to busy clinical staff and develop initial guidelines for a future system. Any system developed using these guidelines could be evaluated in future studies.

### Measuring Activity in a Joint Replacement Population

An objective method of activity assessment—step counting—has been accurately used to monitor changes in gait and activity in musculoskeletal disorders and diseases affecting gait, including hip and knee arthritis [18,19]. The current objective method used to measure function is accelerometry via wearable sensors. These are inexpensive and easy to wear. However, the data currently derived from these sensors have some limitations, particularly when measuring complex activities and movements that are common in activities of daily living [17,20]. To capture the daily variation in a patient’s functional abilities in their real living environment, it is necessary to move to automated measurement in the patient’s home as well as toward analysis techniques that more directly reflect performance in activities of daily living. For example, cameras can be used to study the kinematics of the transition from sitting to standing [21].

The Sensor Platform for Healthcare in a Residential Environment (SPHERE) Interdisciplinary Research Collaboration has developed a technology comprising an integrated platform of low-power sensors that can measure information continuously about the home (eg, temperature, energy consumption, and humidity) as well as information about people in the home (eg, location, how active they are, and extent of movement) and their health-related behaviors [22]. Data capture has been demonstrated over months or years [23], and so the continuous time-series data collected by SPHERE offer a potentially useful source of data to supplement conventional methods such as PROMs.

This study considers real time-series data generated by SPHERE systems monitoring patient activity in the home before and after total hip or knee replacement. The types of data available from the SPHERE system in each home include metrics derived from Bluetooth-based indoor localization of the patient [18], continuous estimation of posture and ambulatory activities using a wrist-worn accelerometer [24], and silhouette data generated using a depth-sensing video camera [25]. Although the overall system was developed by SPHERE in the absence of equivalent commercial systems, the capabilities of such a system would readily be within the reach of several companies in the consumer *smart home* market. The costs of systems of this kind vary according to implementation decisions as different use cases may benefit from the deployment of different sensors. The patient burden is likely to be minimal once the system is successfully in place. In comparison with cross-sectional PROMs, the costs of continuous time-series data monitoring lie primarily in maintenance following initial installation, and hence, time-series approaches may be more practical for longer-term observation of patients’ symptoms. Therefore, the findings of this study are a good guide to the strengths, weaknesses, and potential clinical utility of a near product.

### Objectives

The main objectives of this study were to (1) introduce surgeons to continuous home data by visualizing time-series sensor data, (2) understand how these data could assist in postoperative clinical decision-making, and (3) identify design recommendations arising from clinician feedback.

The study departs from previous literature on the use of data in clinical decision support, which is largely focused on data from clinical environments such as intensive care [26,27]. To date, studies that have presented data from community settings to surgeons have focused principally on manually clinician-reported data [28] and laboratory outcomes [29], such as those commonly stored in electronic health records, or patient self-reported data [30] such as PROMs [31]. Where sensor data are sampled, this is often at a relatively low sample rate (eg, a daily measurement or 12 measurements per day) or over a relatively short period, from a few minutes [32] to a week or month [33]. Herein, we consider the challenges of how busy surgeons would make sense of thousands of data points a day over periods of up to 3 months—as would be easily within the capability and requirements of a home-based sensor system [34,35] monitoring recovery from major surgery [36].

## Methods

### Participants and Recruitment

From October 2018 to May 2019, orthopedic surgeons at a hospital in South West England, United Kingdom, were invited to take part in a focus group study. Participant demographics were collected (sex and level of experience performing hip or knee replacement surgical procedures). Identification of potential participants was conducted using convenience and snowball sampling. During this process, surgeons known to the study team were asked to identify other potential participants. Participants were initially screened against the inclusion criteria (a minimum of 2 years of experience performing total hip and knee replacement procedures). The exclusion criteria were a lack of experience in joint replacement specific to the hip or knee. Potential participants were emailed invitations, and those who agreed to consider taking part were invited to attend focus groups. Individual interviews were offered if the focus group timings were not suitable. Those who were contacted were also asked to nominate other potential participants—a snowball sampling approach.

A total of 9 participants (surgeons who were either working as consultants or registrars [residents]) took part. Several potential participants declined because of their clinical workloads and time constraints. At the start of each focus group, the study was discussed with the potential participants, who were invited to ask any questions about the study. Before the focus group started, they provided their written informed consent to participate, including to the publication of anonymous quotations. Focus groups were held at a clinical research center on the same site as the hospital to make it as straightforward as possible for busy surgeons to attend [37]. Face-to-face interviews at the surgeons’ places of work were offered where attendance to the focus group was impractical because of time or distance. In total, most of the surgeons (7/9, 78%) attended 3 focus groups, and 22% (2/9) attended one-to-one interviews. The sample size was considered adequate as enough information was collected to clearly demonstrate concepts or ideas related to the topic addressed and with sufficient repetition of those concepts [38].

### Ethics Approval

Ethics approval was provided by Southwest – Central Bristol National Health Service (NHS) Research Ethics Committee (17/SW/0121) on June 22, 2017.

### Topic Guide and Procedure

A structured topic guide (Multimedia Appendix 1) was developed by the research team, which comprised an interdisciplinary group of health researchers and psychologists (SG and RGH), orthopedic surgeons (AB and MW), data scientists (IC, HS, ET, MP, AM, MH, and PF), and a translational statistician (AJ).

In part 1, a scenario (Textbox 1) was used as a tool to explore the current clinical systems in orthopedic care.

**Figure 1:**
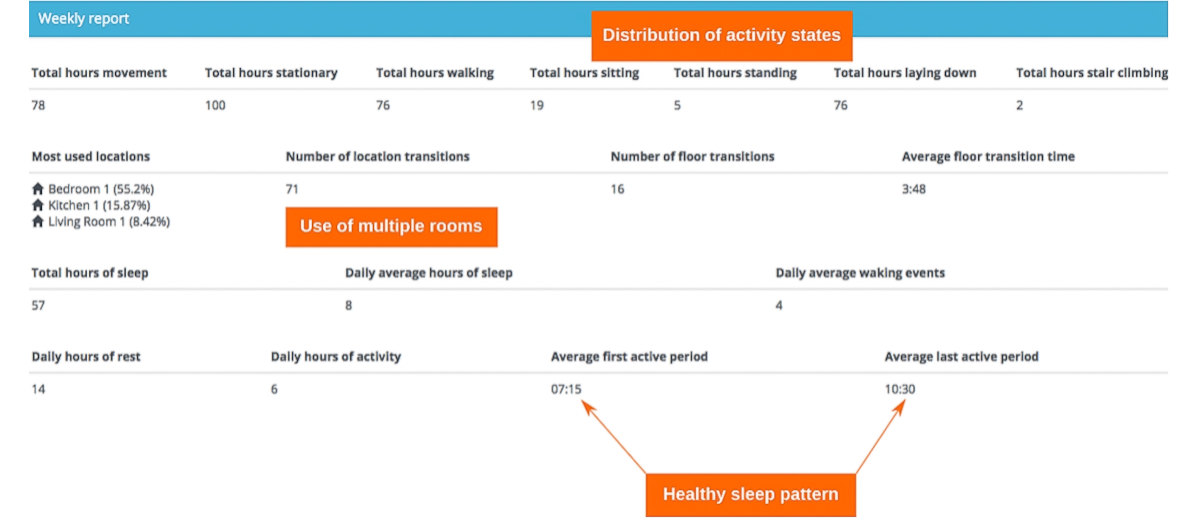
Visualization 1—a screenshot of a dashboard displaying summary statistics, including activity, location, room transitions, floor transitions, and sleep routine.

**Figure 2:**
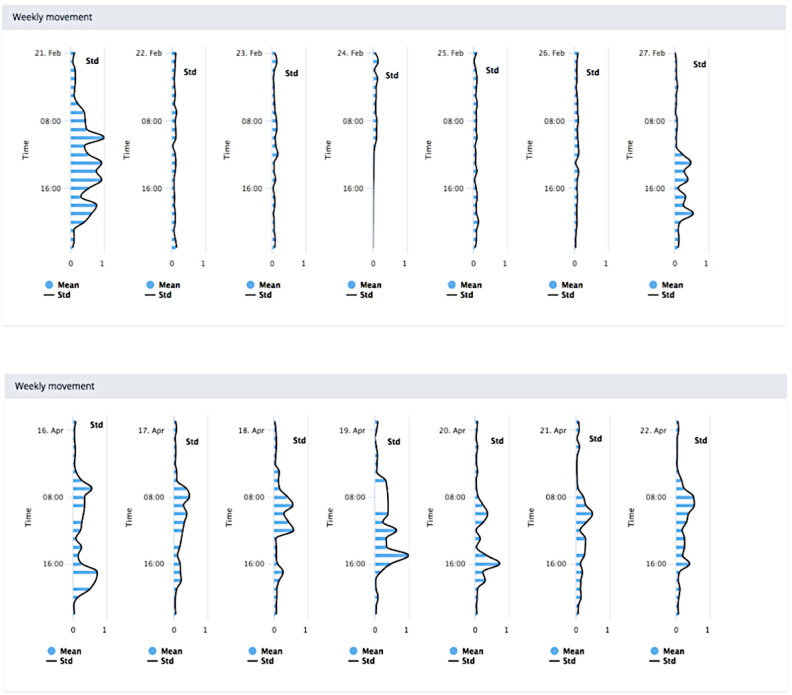
Visualization 2—activity levels of a patient recovering well after surgery.

**Figure 3:**
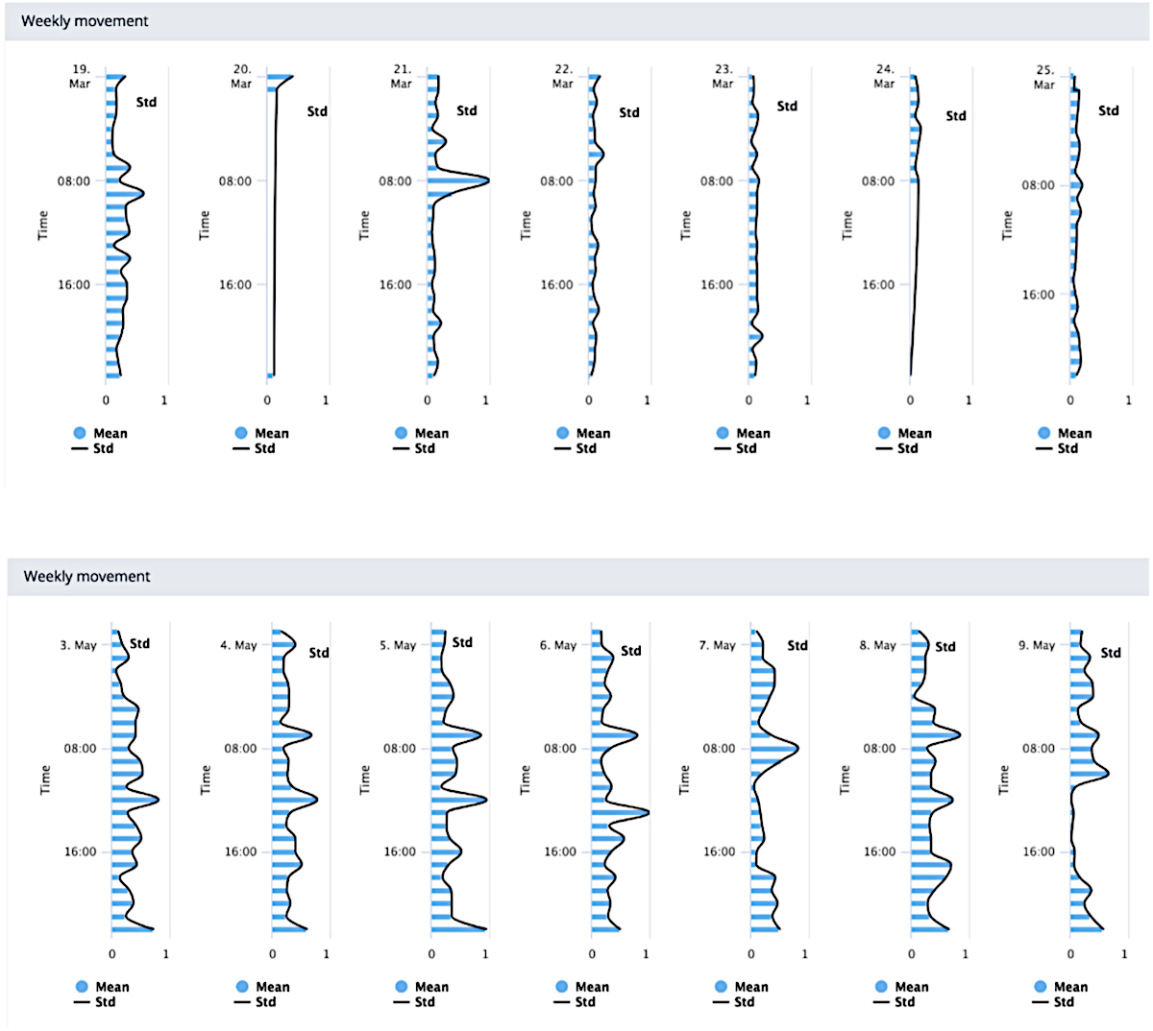
Visualization 3—activity levels of a patient recovering poorly after surgery.

**Figure 4:**
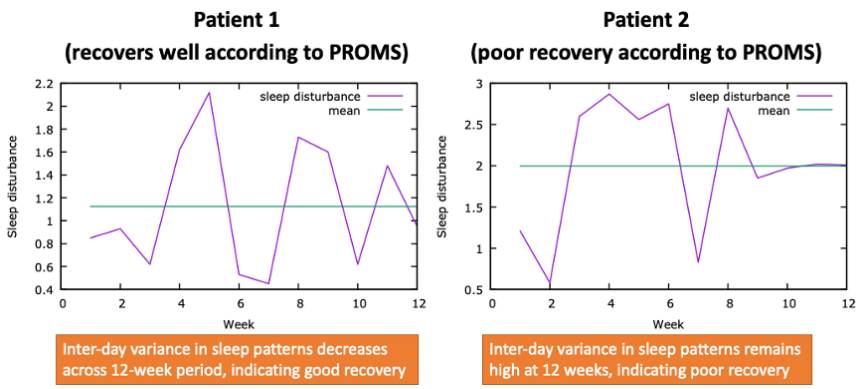
Visualization 4—sleep trend data. PROM: patient-reported outcome measure.

**Figure 5:**
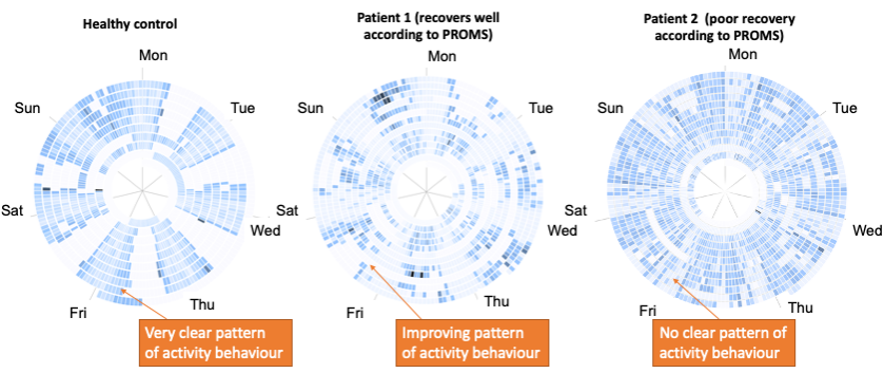
Visualization 5—long-term interpatient comparison of movement. PROM: patient-reported outcome measure.

**Figure 6:**
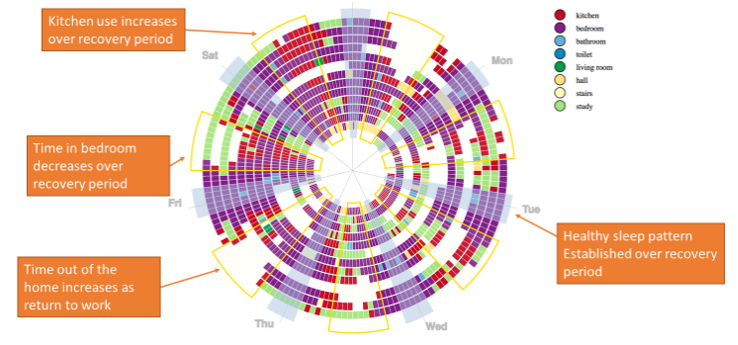
Visualization 6—indoor location data.

**Figure 7:**
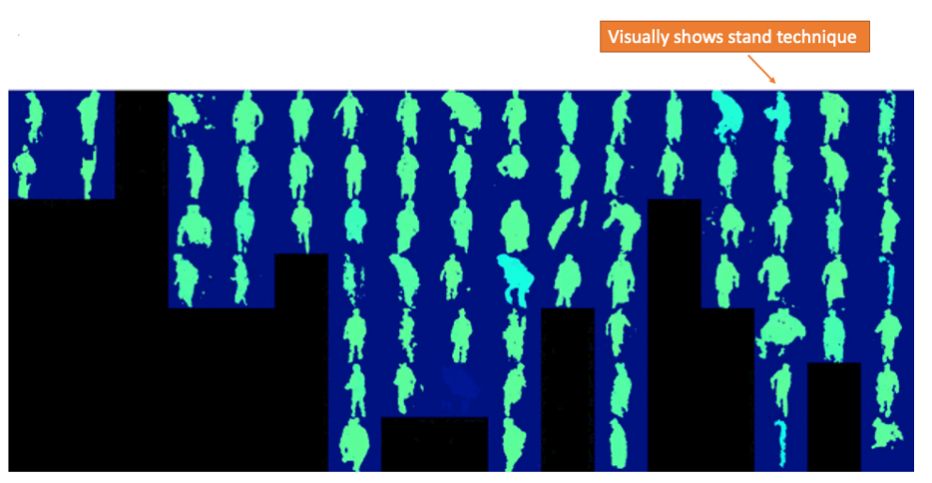
Visualization 7—movement data in long-term sit-to-stand transitions over 16 weeks.

**Figure 8:**
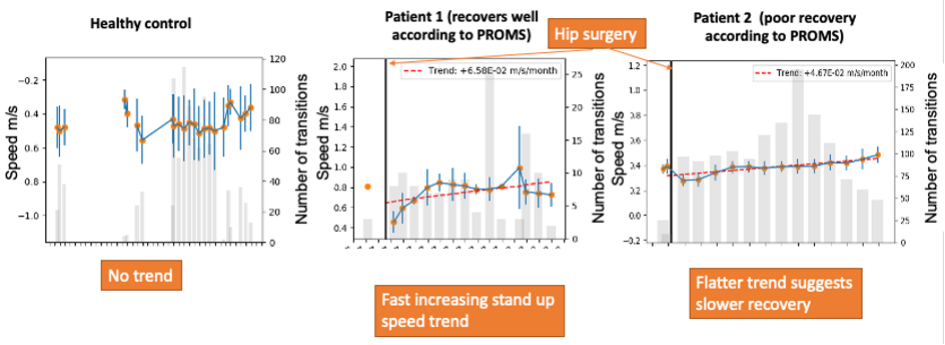
Visualization 8—trends in sit-to-stand speed. PROM: patient-reported outcome measure.

**Figure 9:**
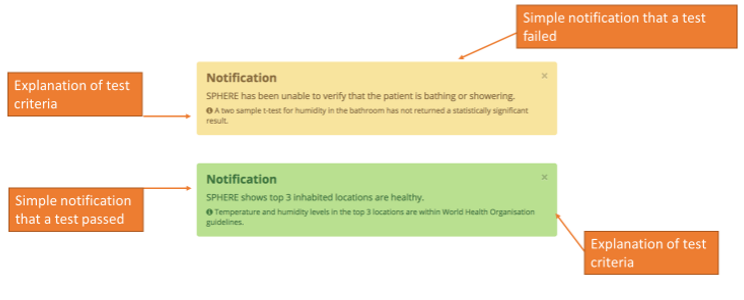
Visualization 9—decision support data notifications and alerts.

In part 2, a series of visualizations (Figures 1-9) were presented based on real participant data from the SPHERE 100 Homes study [23] (in which the system was deployed in homes of the general public) and the Hip and Knee Study of a Sensor Platform for Healthcare in a Residential Environment [39] (in which the same system was deployed in the homes of orthopedic patients).

**Scenario 1—Joyce (aged 63 years).**

- Joyce is a 63-year-old lady who lives in a large three storey house with her daughter and daughter’s fiancé. Joyce is a self-employed therapist and runs her practice from her home. She has a second part-time role at the local University as an administrator.
- Joyce previously had trouble walking distances. Because of a limp she uses a walking aid at times and reports significant hip pain.
- Joyce has recently had her left hip replaced.

Many metrics can be generated using home sensor data. For the purposes of this study, a series of target metrics were generated based on the literature on hip and knee studies. Metrics referenced in manually administered survey instruments such as the OHS and OKS [10] and the Pittsburgh Sleep Quality Index [40] were considered useful targets. Once this step was complete, a series of sample visualizations was generated using these metrics. These visualizations were first proposed and improved over multiple discussions and careful analysis of real patients by members of the Hip and Knee Study of a Sensor Platform for Healthcare in a Residential Environment—mainly data scientists and health researchers. After several iterations, the resulting visualizations were used as examples to provide during the focus groups with clinicians. The detailed rationale behind the development of these figures has been published separately [24]. The use of realistic (eg, noisy and incomplete) prototype data from real homes and real patients was considered desirable throughout this study to ensure that the feedback was related to the characteristics of achievable systems that could plausibly be developed for clinical use in the future.

### Scenario-Based Exploration

An action research approach was used in which participants were seen as able to identify value in context when encouraged to take initiative and identify possibilities for improvements [41].

Participating orthopedic surgeons were presented with a fictitious but realistic orthopedic patient scenario (Textbox 1); the narrative nature of the scenario approach is known to be a useful tool in the design process [41]. Surgeons were asked to focus on the 6- to 8-week postoperative consultation for this hypothetical patient. This creates a familiar and meaningful context [42] in which they are well placed to imagine whether new forms of data would assist them in carrying out their professional responsibilities.

To maximize discussion and allow participants to write thoughts and views independently, participants were provided with printouts of presented visualizations for use within idea generation sessions.

### Focus Groups and Interviews

A total of 3 focus groups (with 2-3 surgeons in each group) and 2 interviews were conducted to explore the data visualizations. Each focus group was facilitated by 2 researchers and lasted approximately 1 to 1.5 hours. The interviews lasted approximately 45 to 60 minutes. The focus groups and interviews were digitally audio recorded and transcribed verbatim.

Part 1 of each data collection phase discussed the scenario (Textbox 1) exploring the assessment of recovery for patients after surgery.

Part 2, led by data analysts (MH and MPN), was a structured exploration in which the surgeons were presented with a selection of visualizations based on data generated from the homes of 2 orthopedic patients who had been recovering from total hip replacement. Participants were asked to consider the use of the visualizations as a way of assessing patient outcome and recovery after surgery. Participants were provided with paper copies of each visualization for any further thoughts or comments that were not captured by discussion—these were reviewed during the coding process.

### Data Analysis

Qualitative data from the focus groups and interviews were analyzed using an inductive thematic approach [43]. The initial labeling generated a list (a “frame”) that was then systematically applied to the data and refined as the analysis progressed [44]. Members of the research team from clinical and nonclinical disciplines were allocated 10% of these transcripts to label independently. After collaborative discussions, further labels were identified, defined, and grouped into themes. This process of investigator triangulation increases internal validity [44]. Excerpts of data were placed on charts according to themes. All data were managed using NVivo software (version 12.0; QSR International).

This qualitative study focuses on the views expressed by the surgeons. Quantitative data were presented to surgeons to elicit those views, but the quantitative data themselves were not the subject of this study. A brief description of the method used to generate the quantitative data and visualizations is provided in the Results section, and the interested reader is referred to the study by Holmes et al [24] for further details.

Once participant feedback was evaluated and themes were identified, feedback was presented to the interdisciplinary research team consisting of researchers, surgeons, machine learners, and interface engineers. This step was intended to facilitate the integration of these findings into future iterations of the sensor and data analysis platform. This step resulted in the development of a series of guidelines that integrate findings from the participants with insights from the interdisciplinary team. This asynchronous codevelopment approach offers an opportunity for participant surgeon feedback and guidance to be made available for future engineering and design processes through the provision of guidelines.

## Results

### Participants and Recruitment

A total of 9 surgeons agreed to participate. Of these 9 surgeons, 6 (67%) were consultant orthopedic surgeons and 3 (33%) were orthopedic registrars (residents)—all the participants saw patients and conducted hip or knee replacement surgery as part of their usual workload, with experience ranging from 2 to 25 years. In total, 22% (2/9) of the participants were female, and 78% (7/9) were male.

### Scenario-Based Exploration

The participants were led in a scenario-based exploration via a fictitious patient scenario, as described in Textbox 1. This generated a stimulated and creative discussion among participants, the outcomes of which are presented in the following section. During data collection and analysis, it became clear that there was a reasonable degree of agreement and repetition in the findings, and sufficient information was deemed to have been obtained in relation to the subject area. In light of this, recruitment and data collection were stopped once 9 surgeons had taken part [38].

### Outcomes From Focus Groups and Interviews

Individual commentary from participants is presented regarding each of the visualizations (Figures 1-9), followed by a series of broader themes from the focus groups and interviews.

Visualization 1 (Figure 1) presented a series of summary statistics calculated using patient indoor location and accelerometer data. These included room-level occupancy data, transitions between rooms, activity predictions generated via machine learning, and actigraphy analysis. The sample data given here were intended to be representative, not exhaustive, and it is possible to identify many other summary statistics that could be relevant.

Participants stated that, although the tabulated data looked very informative, rapid extraction of relevant information was not straightforward given the constraints of a busy 10-minute clinic consultation. Population norms or other references would be required to assess any change or improvement. Visuals would be improved if areas were highlighted to provide a focus for surgeons during a consultation:

So I don’t know where the mobility is on total hours walking, 76, I don’t know what that means, is 76 a lot, is [it] not a lot? Obviously if you had pre-op and post-op data then that’s great because you could get the data just to show you which are better, worse, whatever, but that I think I wouldn’t look at because it would be too hard to navigate.

#0046

Participants reflected on how current methods of assessing patient health outcomes using the OHS or OKS lacked some of the valuable temporal information contained within the SPHERE time-series data. It was suggested that this insight into function over time could help them better understand the recovery process.

Most participants (7/9, 78%) suggested that the existing routine (face-to-face) clinic follow-up appointment presented a similarly rich opportunity for assessing recovery through movement. See the Themes Arising From Data Analysis section for discussion of this point:

So I think actually the bits that I think are important on here all come back to Oxford Hip Score. So people getting up and down stairs, people on the move, people sleeping you know, these are all things that are kind of covered in one way and that, but this [the sensor data] gives you more detail, it’s not just “Yes, good, very good” or whatever. So for me I think, although like the moving one [visualization 7] is quite cool...I don’t see how me looking at that no matter how many hours I had to look at the patient, that when they come in and they stand up and sit down I’ve made my judgment whether they’ve got a problem or not.

#0050

I suppose I’m expecting a patient to be compared against other patients that I’ve seen before. Usually, when I see them, the first thing I do is watch them walk. I watch them walk into the consultation room and check whether or not they’re using walking aids. I then take a good history about how their recovery is going and whether that’s meeting their expectations as well as mine.

#0051

Visualizations 2 and 3 (Figures 2 and 3) illustrated the recovery of 2 patients over weeks 1 and 6 after surgery. The top and bottom figures correspond to weeks 1 and 6, respectively. Patient 1 was an example of a “good recovery,” and patient 2 was an example of a “poor recovery.” “Good” and “poor” recovery were determined descriptively from analysis of PROM data on function and sleep and qualitatively from interviewing the patients, which were data collected as part of the wider program of study [45,46].

The visualizations made use of accelerometer magnitude data, preprocessed to establish the SD, which is an approximate measure of physical activity [47]. These data were then visualized as a series of horizontally stacked axes, each representing a day of the week. Data for 1 week were available in each chart. Stacked charts are a commonly used approach to time-series visualization, with known limitations; notably, line length is visually easier to compare than position [48], and hence, comparison of multiple series is challenging using this type of chart.

Some general suggestions were offered to improve visualization 2. Displaying the mean value of that week rather than daily values was suggested as easier to use:

Because if you put all that together you would get a trace that resembles that [visualization 2/3] and you’d be able to say straightaway that at six weeks, they’re doing great and then at one [week], they’re not.

#0046

However, it was noted that striking the right balance of information using the mean would be a challenge:

The problem is, if you average everything out, then you lose the detail don’t you...but if you present all of the detail, then it becomes impossible.

#0046

Adding a reference value was suggested by some as useful within the weekly charts to demonstrate a “typically” poor recovery and where the patient sits in relation to that. Visually representing this recovery process for patients was again considered a better outlet for a conversation regarding the expectations of surgery:

I guess in the second slide [visualization 2-3] if you were able to put some points to say, this is low activity at time of sleep which is what you’re expecting...

#0051

However, there was a level of disagreement between some surgeons about using population-based comparators with patients, that is, comparing a patient’s data with population norms or averages:

No I think it would depersonalize it in my opinion...Your [the patient’s own] starting point...may be very, very much lower than another patient. So I would just work on an individual improvement.

#0050

The assessment of sleep patterns across a 12-week period is beyond the conventional self-reported assessment of sleep. Within these focus groups, participants viewed long-term changes in sleep as a new approach to understanding recovery. However, as it was an unfamiliar metric, there were varying opinions about how useful this could be for making clinical decisions. On the one hand, it may be helpful for more tailored advice:

Postop [after surgery] we tell them, they’ve got to sleep on their back and most of them have got a bad back, and they hate it, so that’s why they’re doing this but this would be so interesting if in time we changed to give them advice to sleep any which way they like...you might notice a real difference.

#0049

In contrast, some felt that the minutiae of sleep were affected by several different factors following surgery and, therefore, could not be assessed or considered alone:

There are so many other factors that are going to affect sleep other than the joint replacement, especially in this particular demographic...There are potentially too many confounding factors in there for us to be able to use it [visualization 4] usefully.

#0051

Visualization 4 (Figure 4) graphically represents intraday variance of sleep patterns drawn from raw actigraphy data.

Visualization 5 (Figure 5) presented a spiral representation of patient physical activity (derived from accelerometer data). Each complete ring represented a week of data—the spiral map was chosen in response to the observation by Weber et al [49] that spiral charts permit visualization of lengthy time series and that the circular representation is appropriate for time series with high periodicity (in this case, weekly periodicity). Each chart represented approximately 2 to 3 months of data.

The color scheme applied identified absence of data as pure white and represented increasingly strenuous activity using darkening shades of blue. Hence, regular and restful sleep patterns were reflected using light blue “striping” through each period of least activity, with the most active periods represented as dark areas. Periods outside the home resulted in white striping (absent data) on the chart as the project did not collect data outside the home. Known limitations of the spiral format include difficulty reading “older” data at the center of the spiral because of the small area of the central cells. Facilitation involved some narrative running alongside the presentation of visualizations 5 and 6, which explained the unusual visualization to participants.

Across the focus groups, opinions varied on the use of these representations.

In contrast to tabulated data illustrated in visualizations 2 and 3 (Figures 2-3), participants felt that this visualization provided a layer of depth to understanding activity beyond a conventional table:

I quite like...[visualization 5] because I think that at least gives you a bit more depth to the data rather than just a bog-standard table form...visually the patient can understand it with a similar explanation.

#0052

...you want it to be as simple as possible, the patients want to see [physical] models, they want to see x-rays, they want to see a simple form of data that shows that they’ve done better, or they are improving.

#0050

Although interesting to some surgeons, most participants (6/9, 67%) thought that simple line graphs would be more user-friendly.

Furthermore, comparing data from week to week seemed to be favored as a tool for discussion with patients, primarily for illustrating any improvements to them:

...again we want to have less explanation to the patient as possible, so something visual that they can see, this was my activity level, a percentage even, pre [surgery], this is what is was post [surgery]...the patients just want to know has it made any difference, has it improved from their pre-operative state?

#0050

Presenting data in this way stimulated new ways of thinking about activity for the participants—specifically, looking at variation in activity levels over time rather than absolute magnitudes:

I think it’s brilliant what it’s capturing in the house!

#0049

Participants indicated that the variability visible in the charts was of interest but that it was difficult to interpret:

This is an unusual way to display data.

#0051

Making incorrect inferences from the data within a consultation was a concern for the participants, and therefore, guidance or training would be needed for surgeons to use these unfamiliar visualizations:

During the investigation with the patient I would not use the wheels [visualization 5/6]...because it would take twenty minutes to explain to them and half of them still wouldn’t understand. It’s quite a difficult concept.

#0049

Nevertheless, there was broad agreement that such visualizations would be useful for surgeons to use ahead of the consultation but not to explain to patients:

I can see that that [circular plot 1 in visualization 5] is regular and yeah, great, and I can see that [circular plot 2 in visualization 5] is somewhere in between...but I wouldn’t be able to interpret what the hell that means.

#0046

But looking at that and understanding it, I like it but having it explained, having that as a visual reference with the patient in clinic, it would take too long.

#0049

Visualization 6 (Figure 6) presented a spiral chart [49] designed on the same principles as visualization 5, representing a summary of room occupancy information drawn from 14 weeks of a patient’s recovery. Estimates of the average least active times (ie, “L5” in actigraphy terms) and most active times in the participant’s day were drawn from actigraphy data [50] and applied to the chart as an overlay to guide the eye to sections that were expected to have similar characteristics. Empty cells indicated that the participant was not present in the home at that time.

Visualization 6 captures trends in participant behavior within the home. Although clinicians thought that it was useful to describe these trends, they also considered that the data were too complex:

The trends are useful, but although you can see the trends on there, they don’t jump off the page.

#0052

However, identifying anomalies or information that conflicted with any predicted outcomes or expectations was useful:

If you saw that and it shows that they’re not spending any time in the kitchen because they’re immobile because they can’t walk, then that’s useful.

#0051

However, the length of time to arrive at these conclusions was an issue in a short consultation per patient:

It’s really interesting but it takes a long time specifically to, I guess, actually put meaning to it...it needs to highlight people that are struggling or not getting on, rather than presenting really intricate data about what they’re doing which is interesting but...I couldn’t sit down and look at that with every post-op patient.

#0046

Sharing this information with the patient was further highlighted as a challenge if patients interpreted it incorrectly:

That is just an absolute bombardment of colour and data to a patient. It would take you ten minutes to explain activity levels, trends, patterns.

#0051

Accurate assessment of activity using concentric circles was also an issue for some clinicians:

I have a little bit of a problem with it being displayed as a circle because the radius of the circle increases. The surface area of each block increases as you go out. I think it looks like that amount of time is less because you’re looking at something closer in.

Participant 1 in 0051

It feels a little bit as though we’re exaggerating the good bits on the outside.

Participant 2 in 0051

Capturing change over time was viewed as an essential component of the data, and this was not met by this visualization:

I mean, largely, our job is looking at change over time. I don’t think you can interpret change over time very easily on that, I would say (Participant 1 in 0051)...I think we want something that quickly conveys the information that is most important to what your clinical decision making will be at that point.

Participant 2 in 0051

The sit-to-stand movement is used to assess patients in clinics and in research. It can be evaluated using a variety of metrics, including the speed of the motion [51]. Figure 7 is a screenshot of a video showing multiple sit-to-stand transitions collected over the progression of the patient’s recovery (this was an early result from the project, and the video has since been much improved). The data are ordered from left to right, with more recent data to the right.

SPHERE does not store videos captured in people’s homes, and hence, the visualizations presented are “silhouettes.” The use of silhouettes was designed to ensure privacy and acceptability (including acceptability to nonpatient household members and visitors to the home), and some similar processing is a likely feature of any commercial product developed for a similar purpose.

Although the moving images were pleasing, participants least preferred this visualization. Most surgeons expressed that this transition is, in most cases, informally assessed by them as a matter of routine as the patient comes into the first follow-up consultation:

If they walk into your room and they can’t stand up or sit down you know the answer. You’ve actually clocked it before they’ve even got into your room because you’ve watched them get up in the waiting room and walk towards you and whether they’ve got sticks and things. So yeah and you don’t really care about the trend, in that one it’s the absolute.

#0052

Participants recognized that visualization 7 (Figure 7) presented an early prototype of movement data display and not a final outcome:

I don’t think there’s enough resolution there to understand the sit to stand process...we’re primarily concerned about are they flexing too deeply in the early phases, are they rotating and you can’t really see rotation there.

#0049

Sharing this information with patients was also problematic:

It’s too many different images flashing at the same time for a patient to make heads or tails of it.

#0050

Visualization 8 (Figure 8) presented the progression of a quantitative sit-to-stand metric—average speed—over several weeks of recovery time. This is automatically extracted from the data presented in visualization 7 and, hence, constitutes a simplified view of those data.

The speed of transitions over time was viewed as helpful, although an extension of this line of inquiry would be to look at daily habits changing over time:

But not necessarily the sit to stand times so much as actually I was thinking “Oh it’d be interesting to know whether they get down the corridor to the kitchen quicker” because most people having a hip replacement will have a seat they sit in during the day and they will go and make cups of tea and it would be interesting to know are they faster at getting to their kettle over six weeks.

#0052

The speed of the sit-to-stand transition over time is an existing objective test that is sometimes used in clinics. Therefore, this measurement is not in itself an advance in the state of the art in assessing recovery. Rather, the innovation in this case is its use as an in-home metric collected daily. Surgeons proposed that a broader range of metrics could be used to account for the observed range of patient behaviors:

Your data over time from sitting to rising [visualization 8] is useful. There are a couple of other tests so that’s very useful, [an] easy graph for patients to understand. But that’s just one specific activity that you’re looking at.

#0050

Yeah, but it’s a pity it doesn’t measure how far they walk, and is it possible to capture activity data outside the home? Because we do get patients who will [unnecessarily] restrict themselves, particularly older patients, doing their exercises in the house and you’ll get patients at week one or week two, [who] are going round the block.

#0049

Visualization 9 (Figure 9) presents a screenshot of a prototype decision support tool. Sample notifications are presented that make use of available environmental and participant localization information (such as that displayed in Figure 6) to generate responses to 2 example tests: patient bathing or showering (top) and the suitability of the environmental conditions within the home in comparison with standard guidelines (bottom). In practice, it is likely that many unitary tests of this nature exist. Hence, to avoid a “busy” interface, the results would be filtered in accordance with clinical decision support recommendations in a real-world context of use [52].

This final visualization stimulated lively discussions in the focus groups and interviews. The presentation of a notification dashboard aligned with several of the clinicians’ previous expectations of how the data might look. Participants consistently mentioned that clinical tools should capture key pieces of information that can be interpreted rapidly and accurately by both the clinician and patient:

I think that’s what most surgeons would use, and they’d have ten minutes in the clinic and they go.

#0049

I really like the dashboard, “your patient can’t sleep,” “your patient can’t do the stairs,” “the humidity suggests they haven’t had a shower for two weeks,” you know, that kind of data is really helpful—“they can’t cook.”

#0046

This is a very good thing, because you don’t want the surgeons interpreting their own way because they might interpret very differently.

#0049

Therefore, the presentation of this visualization best met the expectations for a tool that could be used in current clinical workflows:

When we go back to that one [visualization 9] that’s quite useful is someone automatically telling us, exactly as you said there, a flag saying “the patient isn’t going out as much as they used to” or “the patient is going out more than they used to, the patient doesn’t appear to be sleeping as much as they used to” or “sleep patterns are still irregular at 6 weeks.” I think those notifications would be good, but I could imagine there being a fairly hefty list of them.

#0052

You know, much as we get ten minutes per patient, you will get the occasional patient who’ll take forty minutes to sort out with a ten-minute slot. And then you’re playing catch up for the next five patients, and that’s the point where you switch to the notification screen, right, is there anything standing out that I need to know about.

#0049

Textual summaries such as these notifications alerting the surgeon to relevant features within the data were perceived as aligning well with a 6-week follow-up consultation appointment routinely offered to patients during which the surgeon could include this information in their existing review procedures. Central to this appointment was the opportunity for surgeons to identify any early warnings of surgical complications from the patient’s perspective:

I think it is useful to have these notifications definitely, the point of the six week follow up is to identify patients who’ve got a problem that we need to do something about and those are principally early infection and dislocation or an early fracture. That’s the point of that six week appointment and so that’s what we need these tools to tell us. Or [alternatively] the other point of it would be do we need to offer more social support or physiotherapy support in this patient’s home to prevent them falling over or help them?

#0051

At six weeks, its actually quite useful because if they’re not actually showering, they might think that’s [be]cause they can’t get the wound wet, patients do have concerns about that.

#0049

Participants broadly liked the idea of a top-level decision-making process integrated within a dashboard, primarily as this removed the need to interpret data for every patient and enabled integration more easily with current clinical management systems:

So you mentioned dashboard, so if I was on BlueSpier [a clinical management system]...if I went in to the patient...if it came up as a red alert, then I’d have a look at it and then go into the data a little bit closer and speak to them about it.

#0046

Some participants (3/9, 33%) felt that similar reports could usefully be distributed in paper form (or presumably by email) to the patient, suggesting that patients may engage with this information outside appointments:

If there was an available one [printout] absolutely, patients would be able to take it home and have a look at it.

#0050

Of the visualizations presented, visualizations 2 to 8 were considered better suited as research tools, whereas the suggested dashboard and notification system presented in visualization 9 (Figure 9) was more appealing for clinical use:

As a clinical tool, I think the notifications are very helpful. I think what would be useful is if you actually provided it to us and gave it to a few surgeons and test it.

#0049

### Themes Arising From Data Analysis

#### Overview

Several general themes were identified common to all visualizations. These were generated by thematic analysis as described in the *Data Analysis* subsection of the *Methods* section. Four themes common across focus groups and interviews were (1) home data represent a more objective measurement of activity, (2) home data provide a stimulus for discussion in a consultation with a patient, (3) there is interest in the use of home data for clinical research purposes, and (4) there is a need to meet clinicians’ requirements in the development of visualizations.

#### Data From the Home Can Give a More Objective Measurement of Activity

Assessment of a patient following surgery is mostly done via a face-to-face clinical appointment approximately 6 to 8 weeks after the operation. In this appointment, through questioning, surgeons routinely assess how patients are getting on at home with their activities of daily living and general independence. Participants widely felt that the SPHERE home data presented an advancement from this current practice:

What would be quite good with this is that you get an objective measure so, you know, can you cook? “Oh yeah, much better.” But you’re not cooking, so it’s not better, maybe physically you can’t cook but the times you have managed to get into the kitchen and it didn’t hurt so you remember it as being better but you’ve only cooked one meal in the week.

#0049

So assessing your patient, you take them on their word really as to how they’re doing. So you ask about how they’re getting on at home, activities, are they still independent, is someone else doing the shopping, can they manage stairs, have they moved, so it’s all those sorts of things which we take on their word. So I suppose this would give you objective information as to whether that’s true, not saying that they’re not telling the truth but it would just give you another side of things.

#0050

The only thing that this offers that we struggle with in clinic is the typical, stoic...farmer that says “No, I’ve not had any help, I’m absolutely fine,” he leaves the room and his wife goes “Yeah, he can’t get to the toilet on his own” and this potentially picks up those problem patients because we do get those patients, not infrequently. And so this is a way of potentially flagging it up if it can do that.

#0052

Some participants expressed that the data could help avoid the common problem that reports by patients of function improvement are often not accurate as they are masked by pain:

I mean if you look at the Oxford Hip Score, 80% of the effects in the Oxford Hip Score are due to pain, so pain dominates in terms of what you see...That’s why they tend to improve [be]cause you’ve reliably improved the pain...but this is the difference, this not reported function, this is real function and the two are quite different. “Can you go up and down the stairs?” is not the same as are you going up and down stairs? That’s what’s useful about this, isn’t it?

#0049

However, a participant identified a dilemma if the home data contradict the patient’s own account, potentially damaging the patient-clinician relationship:

You can’t break that trust that you have to have, if someone says this is what I do, then I have to take that at face value, regardless of whether I believe it or not.

#0046

#### Data From the Home Provide a Stimulus for Discussion in a Consultation With a Patient

Although participants indicated that visualizations 4 to 8 were suitable only for use by clinicians, some visualizations were considered to be a good basis for discussion with patients:

The other thing that I think it would be really useful for is providing information to patients after the operation. So you could monitor a cohort of total hips, total knees, hip fracture, different patients and say “We can expect that your sleep will have returned to normal after eight weeks” or “You will be leaving the house more back the way you were at six weeks” and that would be really useful. So there are some things we get from patients and we tell them that we’re told that on average you get back to bowls [note: the sport of lawn bowls] at six weeks, but actually having a bit of an evidence base to say “...most [patients] were sleeping through the night by six weeks.” That would be a nice thing to be able to say to patients.

#0052

If they say, “I’m not sleeping well,” and you still look and you say, well although you’re not sleeping perfectly, you’ve definitely improved over the last six weeks—do you see what I mean? [Be]cause they don’t always remember that.

#0049

Surgeons felt that it would be feasible for them to use a decision support system in consultations with patients. Given the inevitable complexity of data derived from people’s daily lives, automated processing of data was preferred over a presentation of relatively raw data. Surgeons found the breadth of possible patient information fascinating. However, many said that the need for speed in their necessarily brief consultations left little room to conduct anything other than the “essentials”:

It’s got huge amount of potential, I just don’t know what to do with all these lovely graphs and figures really.

#0052

I think it’s fascinating to see and I think but the reality at the coalface is that in a clinical situation, you just need to do essentials as quickly as possible. I’m struggling to see how that could happen in the ordinary, everyday situation because in this early phase, patients’ recovery trajectories will vary very much...during this early phase, depending on their co-morbidities and everything else, there’s a very different speed of achieving certain milestones.

#0051

I do think that there is a time element there when you’re using the data...if you had a summary page and that was compared to what a normal recovery would be, like a traffic light system. It’s way too much information to process in a clinic.

#0051

Sadly I can’t get past the fact for routine follow up of post-op [hip replacement] patients, we’re already cutting back how many we see and what we do, because they all tend to do so well and so giving us more information is probably not helpful.

#0050

#### Use of Home Data for Research Purposes

A possibility was that the data could be developed into outcome measures for research purposes, with the overall aim to be able to consult such information when addressing individual patient cases:

Certainly from a research point of view if you’re wanting to follow something up like a new hip prosthesis and you wanted to know whether this was making any difference in this early phase...this could be very useful in supporting that.

#0051

Using the data as an outcome measure for research was felt to have considerable potential:

I think the power of this is on a clinical basis, we could do more pilot stuff, you can correlate that with your interviews with the patients.

#0046

#### Meeting Surgeons’ Requirements in the Development of Visualizations

Most participants (8/9, 89%) identified concerns regarding existing visualizations and proposed a way to address them. Challenges included the difficulty of representing large amounts of time-based data without losing detail, accessibility of visualizations to patients, the time required to interpret the visualization, and the provision of excessive detail. To address these issues, surgeons suggested that goal-focused visualizations that solve a small number of competency questions would be of value. For instance, charts showing “a simple form of data” could more easily support clinical evaluation of “one specific activity.”

## Discussion

### Principal Findings

On the basis of an action research approach [41], this paper reports the findings of scenario-based focus groups and interviews. This study aimed to provide insights into the presentation of time-series data as a way of assessing recovery after surgery and to what extent the data supported clinical workflows. Participants generally noted that the data offered a more objective assessment of patient recovery than current methods used in their clinical practice.

Of the visualizations presented, a dashboard comprising specific notifications and alerts seemed to be the best fit for existing workflows. Automation of clinical decisions based on “moment-to-moment quantification of individual level data” [53] and rapidly condensing large amounts of data into meaningful information aligned with the 10-minute appointment time that NHS surgeons have with patients at follow-up.

The tabular and circular data visualizations spanning longer periods were considered useful by surgeons for identifying trends and changes ahead of the consultation. Furthermore, the granular detail of patients’ movement trajectory immediately before and after surgery was considered useful within a consultation, in which the surgeon and patient could address expectations of outcomes after surgery and longer-term follow-up.

It was noted that such discussions would require assurance of sensitive and accurate interpretation of any data beforehand to avoid any negative impact of patient engagement with the data. Furthermore, it would be necessary to decide whether to measure the patient’s progress in absolute terms with reference to a population mean or purely relative to their own initial baseline.

Surgeons are not accustomed to visualizing and conceptualizing time-series data from the home, and as with any new form of clinical data, undoubtedly training would be a prerequisite for the introduction of this type of data into clinical practice. The participants in this study were interested in the complexity of the granular data and were aware that insights would be lost if they were summarized or averaged. However, they had not been provided with professional development training in interpretation of the data. It is reasonable to suppose that training and familiarity would unlock more of the value in the data and lessen some of the legitimate concerns about the data being confusing or time-consuming to use. The challenge of finding intuitive ways of presenting weeks of continuous data to clinicians for use in a 10-minute clinical appointment would be a good area for future research.

### Comparison With Prior Work

The UK National Joint Registry recently introduced a patient decision support tool that aimed to enhance patients’ understanding of their own risks and the potential benefits of having joint replacement surgery [54]. Innovative tools may empower patients to have informed conversations with their physicians about treatment options, and such tools can support evidence-based choices, moving closer toward personalized medicine. Our findings suggest that a clinical decision support system that tracks and interprets activity at home could supplement such information, further enhancing a patient’s choices about treatment options and postrecovery options after surgery. Furthermore, by collecting data before and after surgery, there is the chance to compare outcomes after surgery with presurgical ability and help in communication about expectations before surgery and whether those expectations have been met.

Clinical decision support systems make use of appropriate data analytics and visualization methods to provide advice and guidance to aid health care providers’ problem-solving and decision-making [55]. Potential benefits of designing clinical decision support systems include improving consistency in decision-making, increasing efficiency, and reducing task interruptions and the corresponding fatigue [56]. A recent randomized controlled trial that evaluated the use of a patient decision aid and preference report (ie, a summary of patient clinical and decisional data) by surgeons performing joint replacement [57] found that this supports shared decision-making between clinician and patient and that there was significant improvement in decision quality when such aids were used. The findings of our study are in accordance with the proposal of increased efficiency to some degree as surgeons thought that although some of the home data would be helpful, it would be unlikely that they would directly reference or share this information in consultation because of the limited time they had with patients.

The wide range of sensors available to patients as wearables or within smart home products can help patients track exercise, sleep, heart rate, and much more. Data collected by such sensors to improve health have been used to help with diagnosis and monitoring in the fields of chronic health conditions [58] and mental health [59]. This study illustrates the potential for home data of this kind to be used to support clinical follow-ups after hip and knee replacement surgery. Our study presents a novel exploration of movement data collected via a platform of sensors for use in orthopedics, aiming to yield new ways of advancing conventional follow-up assessments following total hip and knee replacement surgery.

### Strengths

A key strength of this study was the use of an action research approach, which included an exploratory phase followed by discussion of a proposed model of data presentation using real patients’ stories. The triangulation of patterns detected in the quantitative data with real patient participants’ accounts from qualitative interviews contributed to a robust analysis of the data with a good degree of accuracy. A qualitative analysis of patients’ experiences has been reported elsewhere [45]. Finally, this study uniquely explores surgeons’ views of data visualization from novel sensing technology, which is not currently commercially available but could be put on the market in the near future if desired. Insights from this study can help inform research and design directions for products in this space.

### Limitations

The sample comprised surgeons from 1 UK hospital and, as such, only reflects experience in 1 setting. Convenience sampling was used, which may limit the ability to generalize from this sample. However, in practice, participants’ background, skills, and experience were heterogeneous, as were their age and sex. The experiences described are likely to be consonant with those in other contexts, and all UK surgeons follow national approaches and training. More importantly, there may be differences between the findings of our study and those that are relevant in other countries; although the surgical procedure is similar in different contexts, patients’ expectations and the resources available to surgeons may vary. Furthermore, in the United Kingdom and internationally, professionals other than surgeons are involved in the provision of care to patients undergoing knee or hip replacement. For instance, specialist physiotherapists are involved in assessments before surgery and provide care afterward. We did not include their professional experience in this study, and this could be a topic of further research; however, in practice, most health professionals face similar challenges related to time pressures on consultations and the need to collect and convey clear and relevant information.

Everyday practice following the COVID-19 pandemic has required adjustment to deal with service backlogs, such as a move toward day case surgery as well as decreasing length of stay and adoption of remote assessment of postoperative recovery status. It is not yet clear to what extent what proportion of sites has moved to this model and what proportion of patients are affected by this change. There is also a move toward patient-initiated follow-up. However, this is at an early stage, and it remains to be seen what the benefits and shortcomings might be for patients and participants.

### Conclusions and Recommendations

#### Overview

In line with an action research approach [41], we propose the following 4 guidelines for further design and development of home activity monitoring systems. Each guideline draws on the findings described previously and was codeveloped by the interdisciplinary group of coauthors in light of the findings. As such, the guidelines consolidate the views of surgeons and the thematic areas developed in this study and provide recommendations for next steps, including how best to support surgeons—or other health care professionals—and how best to design and deliver a user-appropriate system. Each guideline reflects the content of more than one thematic area. Our aim was to build on surgeons’ views to provide concrete recommendations to support future developments in the collection, visualization, and use of data on recovery or other health changes.

#### Guideline 1: Minimize the Risk of Misinterpreting Data

Surgeons demonstrated consensus on the importance of reducing the risk of misinterpretation of data and the associated variability of interpretations between surgeons. To minimize the risk of misinterpretation, clear summary statistics are recommended. Explainable design principles appropriate for each visualization or presentation of data should be applied to clarify the meaning and limitations of the data and the associated findings. It would be misguided to promise absolute objectivity as the activities of data acquisition, data analysis, and machine learning frequently result in the reproduction of bias present in source data or in the unconscious predispositions held by data analysts themselves [60].

#### Guideline 2: Express the Level of Confidence in the Data

Surgeons expressed a preference for simple and unambiguous metrics. However, electronics and sensor systems in the home inevitably experience many challenges to reliability, such as device failure or wireless network failures; therefore, data from such an uncontrolled environment must always be interpreted with caution, and a level of confidence would need to accompany any data analysis.

#### Guideline 3: Improve Familiarity With Time-Series Data

Exploratory methods of accessing big data are a poor fit with constraints on surgeons’ time. Efficient, rapidly accessible representations of home data requiring minimal expert knowledge are recommended in the first instance. For example, data summarization can facilitate the interpretation of complex data, removing outliers and supporting existing clinical consultation activities. The 2019 Topol Review [61] indicates that training and digital literacy are key to making the most of digital health technologies, particularly artificial intelligence and machine learning. Identifying an understanding of confidence and probability is a necessary prerequisite for interpreting these data and is a required skill. We suggest that familiarity with time-series representations of data acquired through training may increasingly be an advantageous skill for clinical purposes.

#### Guideline 4: Consider the Impact of Patient Engagement

Data are of interest to surgeons as a resource that they can use to assist in their communications with patients. Future developments such as interfaces that support patients in examining their own data may offer a level of empowerment. Greater patient empowerment is positively associated with adherence to treatments and improved outcomes [62]. It also supports the UK NHS commitment to person-centered care, in which patients are encouraged to be actively involved in their own care [63]. Therefore, patient-centered design practices are a substantial component in the development of systems that use home data to support patient-clinician interactions. The time constraints experienced by surgeons limit their opportunities to have direct overview of time-series home data. A patient-centric approach could support patients in monitoring changes in their own condition, potentially facilitating conversations with clinicians. Finally, the schedule by which surgeons or other clinicians review data is not a close fit with the potential for “just-in-time” alerting systems, suggesting that some of the potential of home data may rely on structural innovation and integration with wider support teams.

## Appendix

Multimedia Appendix 1 Topic guides used during this study.

## Abbreviations

NHS: National Health Service
OHS: Oxford Hip Score
OKS: Oxford Knee Score
PROM: patient-reported outcome measure
SPHERE: Sensor Platform for Healthcare in a Residential Environment
