# Supplementary material for: Toward Enhanced Clinical Decision Support for Patients Undergoing a Hip or Knee Replacement: Focus Group and Interview Study With Surgeons
Source: JMIR Perioper Med. 2023 Apr 24;6:e36172. doi: 10.2196/36172 (PMC10167586; doi:10.2196/36172)
Supplement: Multimedia Appendix 1 [file periop_v6i1e36172_app1.pdf]

Hip and KnEe study of a Sensor Platform of Healthcare in a Residential Environment (HEmiSPHERE)

Topic Guides (Patient)

Box-1 Topic Guide – Pre-Surgery (Patient)

- Case history to referral for surgery
- Household constitution
- Experience of health technology (home, wearable, apps)
- Current experience and future expectations of mobility and function
- Preparations in the household for surgery

Box 2 – Topic Guide – Post-Surgery (Patient)

- Experience of aftercare post-surgery
- Experience of living with SPHERE technology
- Relate data from individual PROMs within the questions?
- Ask about the adequacy of information received about SPHERE technology
- Explore how initial expectations of living with the SPHERE technology compares to those after surgery

Box 3– Topic Guide – Post Operative (Health Professional)

- Primary aim of the focus group will be to engage in a stimulus based discussion of the two data sources (PROMs and output of the SPHERE sensors)
- Relevance of each sources to clinical decision making
- Compare the two data sources on accuracy, reliability and usefulness
